# Identification of rare DNA sequence variants in high-risk autism families and their prevalence in a large case/control population

**DOI:** 10.1186/2040-2392-5-5

**Published:** 2014-01-27

**Authors:** Nori Matsunami, Charles H Hensel, Lisa Baird, Jeff Stevens, Brith Otterud, Tami Leppert, Tena Varvil, Dexter Hadley, Joseph T Glessner, Renata Pellegrino, Cecilia Kim, Kelly Thomas, Fengxiang Wang, Frederick G Otieno, Karen Ho, Gerald B Christensen, Dongying Li, Rytis Prekeris, Christophe G Lambert, Hakon Hakonarson, Mark F Leppert

**Affiliations:** 1 Department of Human Genetics, University of Utah, Salt Lake City, UT, USA; 2 Lineagen, Inc, Salt Lake City, UT, USA; 3 Center for Applied Genomics, The Children’s Hospital of Philadelphia, Philadelphia, PA, USA; 4 Golden Helix, Inc, Bozeman, MT, USA; 5 Department of Cell and Developmental Biology, School of Medicine, University of Colorado Anschutz Medical Campus, Aurora, CO, USA; 6 Department of Pediatrics, University of Pennsylvania School of Medicine, Philadelphia, PA, USA

**Keywords:** Familial autism, Haplotype sharing, DNA sequence variants, Case/control study

## Abstract

**Background:**

Genetics clearly plays a major role in the etiology of autism spectrum disorders (ASDs), but studies to date are only beginning to characterize the causal genetic variants responsible. Until recently, studies using multiple extended multi-generation families to identify ASD risk genes had not been undertaken.

**Methods:**

We identified haplotypes shared among individuals with ASDs in large multiplex families, followed by targeted DNA capture and sequencing to identify potential causal variants. We also assayed the prevalence of the identified variants in a large ASD case/control population.

**Results:**

We identified 584 non-conservative missense, nonsense, frameshift and splice site variants that might predispose to autism in our high-risk families. Eleven of these variants were observed to have odds ratios greater than 1.5 in a set of 1,541 unrelated children with autism and 5,785 controls. Three variants, in the *RAB11FIP5, ABP1,* and *JMJD7-PLA2G4B* genes, each were observed in a single case and not in any controls. These variants also were not seen in public sequence databases, suggesting that they may be rare causal ASD variants. Twenty-eight additional rare variants were observed only in high-risk ASD families. Collectively, these 39 variants identify 36 genes as ASD risk genes. Segregation of sequence variants and of copy number variants previously detected in these families reveals a complex pattern, with only a *RAB11FIP5* variant segregating to all affected individuals in one two-generation pedigree. Some affected individuals were found to have multiple potential risk alleles, including sequence variants and copy number variants (CNVs), suggesting that the high incidence of autism in these families could be best explained by variants at multiple loci.

**Conclusions:**

Our study is the first to use haplotype sharing to identify familial ASD risk loci. In total, we identified 39 variants in 36 genes that may confer a genetic risk of developing autism. The observation of 11 of these variants in unrelated ASD cases further supports their role as ASD risk variants.

## Background

Twin and family studies clearly demonstrate that genetics plays a major role in the etiology of ASDs [1-7]. However, family-based approaches using sib-ships or nuclear families have not resulted in the identification of genes or variants that explain a significant portion of the affected population. Similarly, genetic linkage studies have identified a number of chromosomal regions that are thought to contain genes that predispose to ASDs, but identification of the relevant gene(s) in these regions has proven difficult.

More recent analyses have revealed that many of the point mutations thought to predispose children to ASDs were not present in either parent, and thus occurred spontaneously in the affected individuals [8-12]. Such *de novo* variants likely account for as much as 20 to 30% of the genetic variation which results in ASDs. Additional cases are likely due to recessive inheritance of non-functional or hypo-functional alleles, but autosomal recessive inheritance is thought to explain only about 1% of autism cases [13,14].

In addition to single nucleotide variants and small insertions/deletions that can be identified by DNA sequencing, larger deletions or duplications (copy number variants, CNVs) have been shown to play a role in the etiology of ASDs [15-27]. Despite the observed inheritance of many ASD predisposition CNVs from an unaffected parent, the lack of extended, multi-generation pedigrees has precluded a comprehensive analysis of segregation of putative ASD predisposition CNVs and single nucleotide polymorphisms (SNPs) and the characterization of other genetic factors necessary for their expression.

The large families available in Utah coupled with the willingness of family members to participate in genetic studies have resulted in the identification of a large number of disease predisposition genes for both Mendelian and complex diseases. The pedigrees used in this study were part of a 70-family linkage study published previously [28] and two smaller studies that evaluated a single extended pedigree in this collection of families [29,30]. In this work, we analyzed members of 26 extended multi-generational ASD families and four two-generation multiplex ASD families by performing haplotype sharing analysis to identify chromosomal regions that might harbor ASD predisposition genes. We then used DNA capture and sequencing of all genes in shared regions and of additional candidate autism risk genes to identify SNPs that might predispose to ASD in these families. These SNPs were analyzed in a large case/control study and for segregation in these families. We also evaluated the segregation of CNVs, reported previously [27], in these families. Consistent with earlier studies, no single locus could account for more than a subset of the affected individuals in any extended pedigree. In particular, multiple potential risk alleles, including in some cases CNVs and SNPs, were identified in an extended pedigree, suggesting that no single variant is the genetic predisposition locus for all affected family members. The data presented here identify several genes that may harbor ASD predisposition mutations, and add to the growing list of genes that are targets for clinical DNA sequencing to aid in the understanding of an ASD genetic diagnosis. These data further suggest that in some individuals multiple genetic variants may be necessary to elicit the observed clinical characteristics and that for a complete understanding of ASD genetics, both sequence variants and CNVs must be analyzed.

## Methods

### DNA samples

A total of 386 DNA samples from 26 extended multi-generation and four two-generation Utah multiplex ASD pedigrees were used in this study. Families were ascertained and recruited using the Utah Population Database (UPDB) as described [28]. Affection status was determined using the Autism Diagnostic Interview-Revised (ADI-R) and the Autism Diagnostic Observation Schedule (ADOS), for both the familial ASD cases and the unrelated ASD cases, as described previously [27]. The average number of affected individuals in each pedigree is 7.9. The pedigrees described here are a subset of those described previously [28]. Pedigree details are shown in Additional file 1: Table S1. The 55 samples used for our previous CNV discovery were included in these families. A total of 9,000 DNA samples previously described in a case/control study [27], including 3,000 individuals with ASD and 6,000 controls, were used to evaluate these variants in a broader population. All samples collected for the work described here were collected under methods approved by the University of Utah Institutional Review Board (IRB) (University of Utah IRB#: 6042–96) or The Children’s Hospital of Philadelphia IRB (CHOP IRB#: IRB 06–004886). Patients and their families were recruited through the University of Utah Department of Psychiatry or The Children’s Hospital of Philadelphia clinic or CHOP outreach clinics. Written informed consent was obtained from the participants or their parents using IRB approved consent forms prior to enrollment in the project. There was no discrimination against individuals or families who chose not to participate in the study. All data were analyzed anonymously and all clinical investigations were conducted according to the principles expressed in the Declaration of Helsinki.

### SNP microarray genotyping

Affymetrix 250 K NspI SNP chip genotyping was carried out on all 386 DNA samples using the manufacturer’s recommended procedure. Genotypes were called by Affymetrix Genotyping Console software using the BRLMM [31] genotype calling algorithm. Only SNPs with call rates greater than or equal to 99% were used for further analyses. SNPs demonstrating Mendelian errors also were identified using PedCheck [32] and were excluded.

### Shared haplotype analysis

We performed shared haplotype analysis on each pedigree to identify genomic regions that have significant sharing among the affected individuals in that pedigree. The HapShare algorithm [33] was used to perform haplotype phasing based on Mendelian inheritance and to identify shared genomic segments. The comparisons included N out of N affected individuals, (N-1) out of N, (N-2) out of N, (N-3) out of N, and so on (See Figure one in reference [33]). In two-generation pedigrees, in some cases co-segregation of haplotypes was observed in all affected individuals analyzed, but the shared regions were large, including up to half of a chromosome. Consequently, shared regions from nuclear families were not selected for sequencing unless they overlapped regions observed in additional families.

### Custom targeted exome DNA sequencing

NimbleGen (Roche NimbleGen, Inc., Madison, WI, USA) custom sequence capture arrays were designed to capture 2,000 base pairs upstream of the transcription start site and all exons and exon-intron boundaries of genes within the shared genomic segments. An additional 23 genes from outside of our haplotype sharing regions were selected from the literature based on their potential roles in autism or neuronal functions (see Additional file 1: Table S2). A total of approximately 1,800 genes were captured. Capture and Illumina DNA sequencing were performed by the Vanderbilt University Microarray Shared Resource facility on DNA from 26 affected individuals from 11 families that showed sharing of genomic segments. Short reads were aligned to the National Center for Biotechnology Information (NCBI) reference human genome build 36 (GRCh36/hg18) and variants were called using the software alignment and variant calling methods described in Table 1[34-36]. Potential variants detected by at least two of the methods were selected for further analysis.

**Table 1:** Sequence alignment and variant detection methods

	**Alignment and assembly**	**Sequence variant detection**
Method 1	Bowtie	Maq
Method 2	MOSAIK	GigaBayes
Method 3	CLC Bio Genomics Workbench (CLC Bio Inc.)	CLC Bio Genomics Workbench

(CLC Bio Inc., Cambridge, MA, USA).

### Variant annotation

*In silico* functional analysis was carried out initially using cSNP classifier, a preliminary program later incorporated into VAAST [37], to classify variants as synonymous, conservative missense, non-conservative missense, nonsense, frameshift, or splice site changes. Later, variants were re-annotated using the ANNOVAR program [38]. The KnownGene and RefSeq gene tracks from the UCSC genome browser were used to annotate functional variants, and the LiftOver tool was used to convert human genome build 36 (GRCh36/hg18) coordinates to human genome build 37 (GRCh37/hg19) coordinates [39,40].

### Custom microarray design and array processing

Design of the custom iSelect Infinium^TM^ II BeadChip array (Illumina Inc., San Diego, CA, USA) including probes for 2,799 putative functional candidate SNPs and 7,134 CNV probes was described previously [27]. The custom iSelect array was processed on 3,000 case and 6,000 control samples at the Center for Applied Genomics at The Children’s Hospital of Philadelphia (CHOP) [27]. The same array was also used to analyze DNA from 196 Utah discovery cohort family members at the University of Utah Genomics Core facility for variant validation and analysis of SNP segregation in families.

### Array data quality control

#### Sample QC

Subjects were withheld from SNP analysis if any of the following were true: 1) subsequent to genotyping, the DNA sample was of apparent poor quality, evidenced by very low call rates (N = 134); 2) the subject was identified as a trisomy-21 (N = 51); 3) the subject was outside of the central cluster of Caucasian subjects identified by principal component analysis (PCA) (N = 903) [27].

Relatedness estimation further indicated that some of the case subjects and controls were part of families with multiple relatives represented in the data. Re-evaluation of family structure in the sample cohorts used subsequently identified additional relationships. Subsequent association tests were therefore conducted using only one member of each known family in order to reduce the possibility of statistical confounding due to relatedness. For these tests, the subject selected from each family was the individual located nearest to the median centroid of the first two principal components. The number of subjects removed due to relatedness was 688. This resulted in a final sample set for association testing comprising 7,326 subjects, of which 1,541 were cases and 5,785 were controls.

PCA was used to avoid artifacts due to population stratification. Principal components were calculated in Golden Helix SNP and Variation Suite (SVS) using default settings. All subjects were included in the calculation except those that failed sample quality control (QC). Prior to calculating principal components, the SNPs were filtered according to the following criteria: autosomes only, call rate > 0.95, minor allele frequency (MAF) > 0.05, linkage disequilibrium R^2^ < 25% for all pairs of SNPs within a moving window of 50 SNPs. Two thousand and eight SNPs, including those used for CNV analysis, were used for the principal component calculations. No genotype data were available for reference populations, as would typically be preferred for making inferences about population stratification. However, a self-reported ethnicity variable was available for most subjects. A plot of the first two principal components shows a primary central cluster of subjects, with outlier groups extending along two axes. These roughly correspond to Asian and African-American ancestry as self-reported in the phenotype data. A simple outlier detection algorithm was applied to stratify the subjects into two groups representing the most probable Caucasians and non-Caucasians. This was done by first calculating the Cartesian distance of each subject from the median centroid of the first two principal component vectors. After determining the third quartile (Q3) and inter-quartile range (IQR) of the distances, any subject with a distance exceeding:

Q3+1.5×IQR

was determined to be outside of the main cluster, and therefore non-Caucasian. Six hundred and eighty-two subjects were placed in the non-Caucasian category. A graphical representation of the results of this PCA analysis were reported previously [27].

#### SNP QC

Prior to association testing, SNPs were evaluated for call rate, Hardy-Weinberg equilibrium (HWE) and allele frequency. All SNPs with call rates lower than 99% were removed from further analysis. No SNPs had significant Hardy-Weinberg disequilibrium.

### Laboratory confirmation of SNPs and CNVs

For molecular validation of SNPs, PCR products were first screened by LightScanner High Resolution Melt curve analysis (BioFire Diagnostics Inc., Salt Lake City, UT, USA) for the presence of sequence variants. PCR primer sequences are shown in Additional file 1: Table S3. Any samples that gave abnormal melt profiles were sequenced using the Sanger method at the University Utah Sequencing Core facility to confirm the presence of a sequence variant. For CNVs, pre- or custom-designed TaqMan copy number assays (Applied Biosystems Inc., Foster City, CA, USA) were used as described previously [27].

### Protein binding assays

All glutathione S-transferase (GST)-tagged proteins were expressed and purified as described previously [41]. To test Rab11FIP5 binding to various Rab GTPases, purified recombinant FIP5(490–653) or FIP5(490–653)-P652L were incubated with glutathione beads coated with GST, GST-Rab11a, GST-Rab4a or GST-Rab3a in the presence of 1 μm GMP-PNP. Beads were then washed with PBS and eluted with 1% sodium dodecyl sulphate (SDS). Eluates were then analyzed for the presence of FIP5 (490–653) by immunoblotting with anti-Rab11FIP5 antibodies. A similar assay also was used to test the ability of Rab11FIP5 (wild-type or P652L mutant) to dimerize.

### Flow cytometry analysis of transferrin recycling

To test the effect of the Rab11FIP5-P652L mutant on endocytic recycling, the transferrin recycling assay was used as described previously [42]. Briefly, HeLa cells expressing either wild-type FIP5-GFP or FIP5-GFP-P652L were incubated with transferrin conjugated to Alexa488. Cells were then washed and incubated with serum-supplemented media for varying amounts of time. The cell-associated (not recycled) Tf-Alexa488 was analyzed by flow cytometry.

## Results

**Figure 1:**
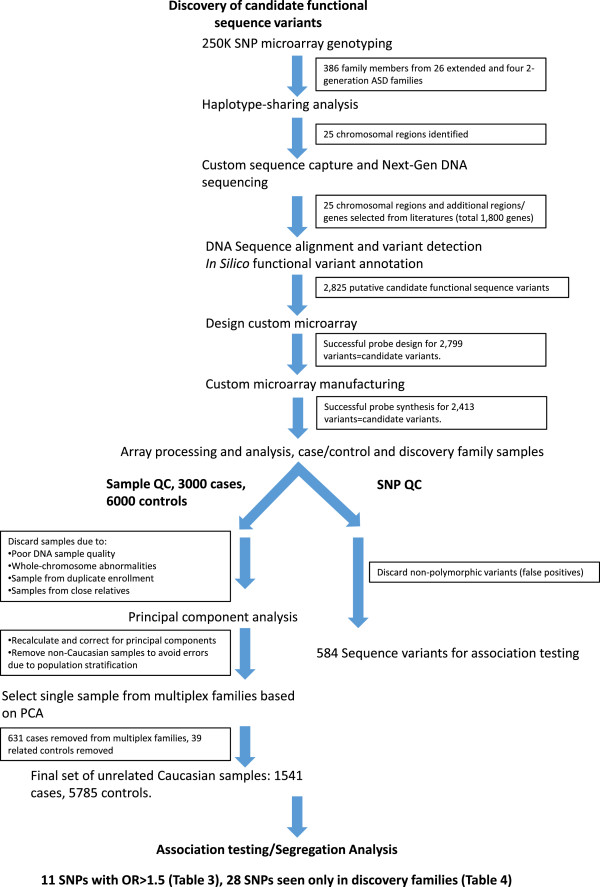
**Workflow for sequence variant discovery and analysis.** Only ethnicity and gender matched, unrelated, cases and controls were used for association testing.

To identify genes that predispose to ASDs in multiplex ASD families, we took a haplotype sharing/custom DNA capture and sequencing approach. We utilized the workflow outlined in Figure 1, first to identify chromosomal regions with excessive sharing among affected individuals in multiplex ASD families. We then used sequence capture to identify potential functional sequence variants in the genes lying in the shared regions, as well as in additional ASD candidate genes. Finally, we evaluated the segregation of those variants in our ASD families and determined their prevalence in a large set of ASD cases and a large set of controls. The details of this process are described below.

### Affymetrix 250 K SNP genotyping and haplotype sharing

SNP genotyping was carried out on 386 DNA samples from 26 extended multi-generation and four two-generation Utah multiplex ASD pedigrees. SNPs with no map location were not included in the analysis. The average call rate was 99.1% for the entire dataset.

We used the HapShare method [33] to identify genomic regions that have significant sharing among the affected individuals in each of the 30 pedigrees we studied. Paternal and maternal haplotypes were determined based on Mendelian inheritance using only informative markers. These haplotypes then were compared among affected individuals within each extended or nuclear family. We selected 18 regions of haplotype sharing based on sharing in extended pedigrees for further analysis. The degree of sharing that we observed among affected individuals and the coordinates of the regions selected for DNA capture and sequencing are shown in Table 2. Two additional regions were selected for DNA capture and sequencing based on a published linkage analysis using an overlapping set of families [28].

**Table 2:** Chromosomal regions selected for sequencing based on haplotype sharing

**Eighteen shared haplotype regions**	**Chromosome**	**Location (hg18)**	**Location (hg19)**	**Affecteds sharing haplotype**
2p14-p12	2	65612029-76349401	65758525-76495893	6 of 6
2q23-q31	2	153638312-174296304	153930066-174588058	6 of 6
2q37	2	231435643-238617145	231727399-238952406	5 of 7
3q13	3	111604019-112685490	110121329-111202800	4 of 7
3q26-q27	3	174594938-185701563	173112244-184218869	4 of 7, 4 of 4
4q28-q31	4	137362554-141629142	137143104-141409692	6 of 6
7p21	7	7381742-11861952	7415217-11895427	4 of 4, 4 of 6
7p14	7	36090817-41521542	36124292-41555017	4 of 7
7q21-q31	7	90511244-107823133	90673308-108035897	5 of 8^a^
7q35-36	7	142750349-151152511	143040227-151521578	4 of 6
12q21	12	76119990-77788028	77595859-79263897	5 of 7
12q21	12	79689788-87939487	81165657-89415356	5 of 8
14q11-q21	14	22912579-45661808	23842739-46592058	3 of 4, 6 of 6
14q32	14	92331535-103509782	93261782-104440029	4 of 4
15q12-q21	15	24339787-43759484	26788694-45972192	3 of 4, 4 of 6, 5 of 8
16q22-23	16	73415053-77780513	74857552-79223012	4 of 7, 5 of 6, 3 of 4
20p11-q13	20	25253250-41225971	25305250-41792557	4 of 7
20q13	20	49062886-57757418	49629479-58324023	5 of 6, 5 of 6

Where multiple numbers are given, multiple families shared overlapping haplotypes. ^a^Indicates a family where a ninth affected individual was later shown not to share the same haplotype.

### Sequence capture, sequence analysis and variant identification

We performed capture and DNA sequencing using DNA from 26 affected individuals from 11 families that showed the best sharing of genomic segments. These samples included individuals from two-generation pedigrees that had shared haplotypes overlapping regions identified in the extended pedigrees. Eight to nine million 36 base short reads were obtained from each sample. The short reads alignment against the NCBI reference human genome build 36 revealed coverage of 86 to 97% of the designed capture area, with the average read depth over the designed capture area of 30 to 47X. Because 1) the capture library was constructed in a directional manner, 2) all capture probes represented the same DNA strand, and 3) the library was sequenced only from one direction, some portions of exons lacked the sequence depth of coverage that we desired. Consequently, there could be additional variants (false negative results) that we did not detect in some of the genes. For example no variants were identified on haplotypes that segregate to all affected individuals in pedigree 10 on chromosomes 2 and 14 (Additional file 2: Figure S1, A and B, Additional file 3: Figure S2). Nonetheless, variant calling using the three methods shown in Table 1 identified over one million sequence variants called by at least two of the three methods. Analysis using cSNP classifier resulted in the detection of 2,825 putative functional candidate SNPs, including 210 nonsense variants, 1,614 non-conservative missense variants, 35 frameshift variants and 966 splice site variants.

We chose to design a custom microarray to evaluate the variants that we identified by sequencing in order to 1) interrogate the entire set of candidate functional SNPs in the discovery families for validation, and 2) to perform a large scale case/control study to determine if any of the variants identified predisposition genes important to the broad population of children with ASD (Figure 1). Following array design and manufacture, probes for 2,413 variants were created successfully. Custom microarray experiments on Utah discovery and CHOP case/control samples revealed 584 out of 2,413 putative variants to be polymorphic. The complete list of polymorphic variants is shown in Additional file 1: Table S4. The remaining array probes did not detect a non-reference sequence allele. These 1,829 variants thus were interpreted to be false positives due to the variant calling and alignment process of single end sequence data.

All autosomal SNP variants were tested for association with autism in our case/control study using an allelic association test. Statistical significance of each was assessed using both Fisher’s exact test and a chi-squared test. The allelic association test detects any significant result regardless of the direction of the effect. Eleven SNPs (see clustering in Additional file 4: Figure S3) were either unique to cases or had odds ratios (minor allele) greater than 1.5 (Table 3). We prioritized the variants observed in our case/control study for additional work based on an odds ratio cutoff of 1.5. We also included variants unique to cases. We chose this approach rather than using *P* values since these variants were too rare to select based on *P* values, and for relatively rare diseases odds ratios are approximately equivalent to relative risk values. In addition, 28 SNPs were detected only in the Utah discovery cohort and not in the CHOP cases or controls (Table 4). We consider these 28 SNPs to be potential ASD risk alleles because a) they are rare or non-existent in the general population and thus could represent ‘private mutations’, b) they may affect protein function, and c) they segregate to one or more children with autism in high-risk autism pedigrees. Thus, we characterize these 39 SNPs, found in 36 different genes, as potential autism risk variants. Each of these 39 variants was localized to our targeted regions (Table 2), and 30 of the 39 variants were predicted to be damaging by at least one program embedded in ANNOVAR [38], including SIFT, Polyphen2, LRT and MutationTaster. Details of the analysis of these variants are shown in Additional file 1: Table S5. All 39 SNPs were further confirmed by Sanger DNA sequencing of PCR amplicons (see Additional file 5: Figure S4, Additional file 6: Figure S5, for sequence chromatograms). The transcripts used for variant annotation are found in Additional file 1: Table S5.

**Table 3:** Sequence variants identified in families and observed in the case/control study

**Variant (Ref/Obs)**	**Gene**	**Coordinate (hg19)**	**Fisher’s exact P**	**Chi-squared P**	**Odds ratio (Minor allele)**	**Odds ratio 95% lower confidence bound**	**Odds ratio 95% upper confidence bound**	**Het. cases**	**Het. controls**	**W-T cases**	**W-T controls**	**Variant**
G/T	RAB11FIP5	chr2:73302656	2.10E-01	5.27E-02	infinite	N/A	N/A	1	0	1,540	5,785	P652H
G/C	ABP1	chr7:150554592	2.10E-01	5.27E-02	infinite	N/A	N/A	1	0	1,540	5,785	R345P
T/A	JMJD7-PLA2G4B	chr15:42133295	2.10E-01	5.27E-02	infinite	N/A	N/A	1	0	1,540	5,785	splice site
C/T	C7orf10	chr7:40498796	4.02E-02	3.13E-02	1.62	1.04	2.53	28	65	1,513	5,720	R288W, splice site
C/T	AKAP9	chr7:91724455	6.62E-02	4.44E-02	3.76	0.94	15.03	4	4	1,537	5,781	R3233C
C/T	HEPACAM2	chr7:92825188	5.84E-02	3.88E-02	1.83	1.02	3.27	17	35	1,524	5,750	G398R
G/T	ALX1	chr12:85674230	2.22E-02	1.49E-02	1.75	1.11	2.77	27	58	1,514	5,727	R64L
G/A	AP1G2	chr14:24035159	1.66E-01	1.35E-01	1.67	0.85	3.30	12	27	1,529	5,757	R99C
G/C	CLMN	chr14:95679692	2.29E-01	2.23E-01	1.67	0.73	3.84	8	18	1,533	5,767	P158A
G/A	MOK	chr14:102749873	1.97E-01	1.55E-01	3.76	0.53	26.67	2	2	1,539	5,783	Q22X
G/A	OIP5	chr15:41611874	3.77E-01	2.53E-01	2.25	0.54	9.44	3	5	1,538	5,780	S165F

Het. Refers to individuals heterozygous for the indicated variant.

**Table 4:** Sequence variants observed only in high-risk ASD families

**Variant (Ref/Obs)**	**Gene**	**Coordinate (hg19)**	**Pedigree structure**	**Tested affecteds in pedigree**	**Affecteds with variant**	**Coding change**	**ESP6500_ALL**
G/A	RAB11FIP5	chr2:73302656	2-Generation	3	3	P652L	
C/G	AUP1	chr2:74756328	Extended	5	1	R90S	
T/C	SCN3A	chr2:165946964	Extended	6	1	E1851G	
T/C	ATP11B	chr3:182583394	Extended	9	2	S451P	
A/G	KLHL6	chr3:183226296	2-Generation	5	4	F154L, splicing	
C/T	AKAP9	chr7:91736684	Extended	7	1	R3832C	0.000154
G/C	PDK4	chr7:95215047	2-Generation	6	3	S381X	
C/G	RELN	chr7:103214555	Extended	7	1	D1499H	0.000231
G/A	DCAF11	chr14:24590630	2-Generation	3	2	G435R	
G/A	RNF31	chr14:24617687	Extended	9	1	splicing	
G/C	IRF9	chr14:24634003	Extended	9	1	R277T	
G/A	SDR39U1	chr14:24909513	2-Generation	6	2	P220S	
T/A	PRKD1	chr14:30095731	2-Generation	3	2	D586V	
C/T	SEC23A	chr14:39545251	2-Generation	3	1	G292D	
G/A	ITPK1	chr14:93418316	2-Generation	5	2	P238L	
G/A	CCDC85C	chr14:99988547	Extended	9	1	R300W	
A/G	C14orf2	chr14:104381450	2-Generation	6	5	I26T	
G/T	TRPM1	chr15:31329966	Extended	5	1	T857K	
T/C	FMN1	chr15:33359761	Extended	9	3	R109G	
G/T	PGBD4	chr15:34395847	Extended	9	4	G372V	0.000231
C/T	JMJD7	chr15:42129054	Extended	9	4	R260C	0.00068
C/T	CASC4	chr15:44620915	Extended	5	1	R139X	
G/C	SPATA5L1	chr15:45695534	2-Generation	5	3	D303H	
C/G	PYGO1	chr15:55839207	Extended	7	1	G92R	
C/G	PRTG	chr15:55916638	Extended	9	2	A999P	
G/A	NUDT7	chr16:77756514	Extended	9	4	R12K, splicing	
G/A	DEFB124	chr20:30053379	Extended	7	4	P49L	0.000154
A/G	EPB41L1	chr20:34809850	Extended	9	1	D733G	

### Segregation of variants in high-risk pedigrees

**Figure 2:**
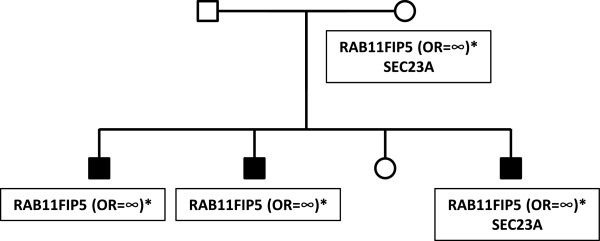
**Co-segregation of a *RAB11FIP5 *variant.** Two-generation pedigree (pedigree 1) with three male siblings affected with autism. Sequence variants identified in the family are shown in the black boxes. Open boxes - unaffected male family members; open circles - unaffected female family members; filled boxes - affected male family members. Odds ratios for the variants observed in the case/control study are shown. Variants with no odds ratio were observed only in high-risk families. All family members were tested for all variants.

**Figure 3:**
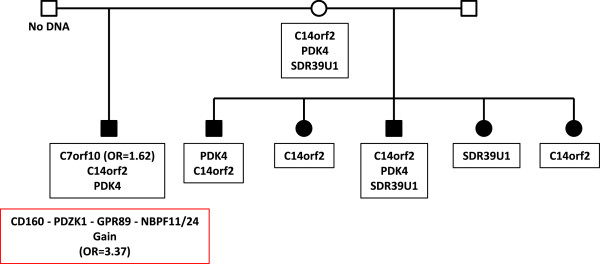
**Segregation of *C14orf2 *variant.** Two-generation pedigree (pedigree 2), with three affected female and two affected male siblings as well as an affected male half-sibling. The *C14orf2* variant segregates to five of six affected children. Pedigree symbols are described in the legend for Figure 2. Sequence variants identified in the family are shown in the black boxes. A CNV found in the affected half-sibling [27] is shown in the red box. Odds ratios for variants observed in the case/control study are shown in parentheses. Variants with no odds ratio were observed only in high-risk families. All family members were tested for all variants unless no DNA was available. Individuals with no available DNA are indicated.

**Figure 4:**
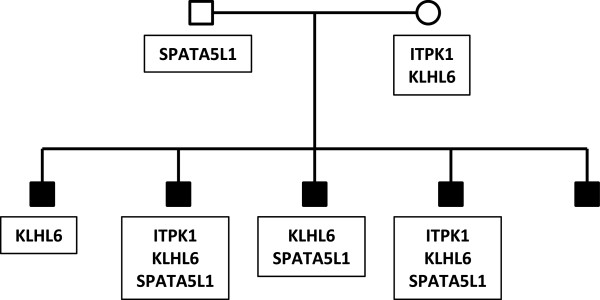
**Segregation of *KLHL6, SPATA5L1, *and *ITPK1 *variants.** Two-generation pedigree (pedigree 3), with five affected male siblings. Sequence variants identified in the family are shown in the black boxes. Pedigree symbols are described in the legend for Figure 2. Variants with no odds ratio were observed only in high-risk families. All family members were tested for all variants.

**Figure 5:**
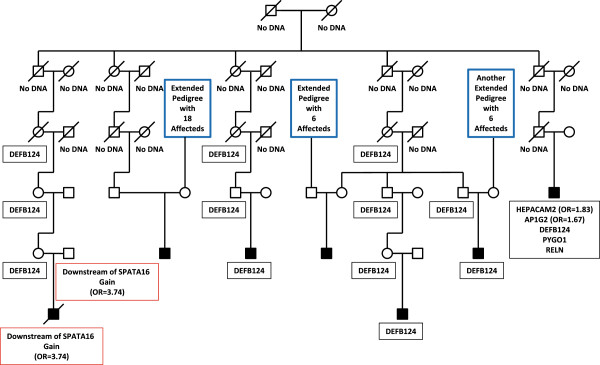
**Segregation of *DEFB124 *variant in a multi-generation pedigree.** Pedigree 4 has seven children affected with autism. Links between this pedigree and other high-risk autism pedigrees are indicated by blue boxes. Sequence variants identified in the family are shown in the black boxes. CNVs inherited by two individuals [27] are shown in red boxes. Pedigree symbols are described in the legend for Figure 2. Odds ratios for the variants observed in the case/control study are shown in parentheses. Variants with no odds ratio were observed only in high-risk families. All family members were tested for all variants unless no DNA was available. Individuals with no available DNA are indicated.

**Figure 6:**
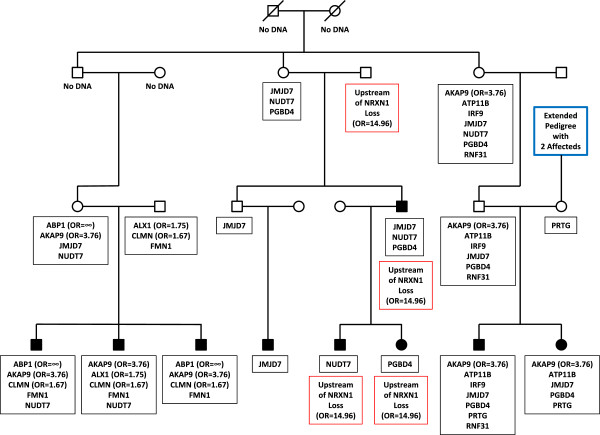
**Segregation of multiple variants including a sequence variant in *AKAP9 *and a copy number variant in *NRXN1 *in a multi-generation pedigree.** Pedigree 5 has nine children affected with autism. A link between this pedigree and another high-risk autism pedigree is indicated by the blue box. Sequence variants identified in the family are shown in the black boxes. CNVs identified in four individuals [27] are shown in red boxes. Pedigree symbols are described in the legend for Figure 2. Odds ratios for the variants observed in the case/control study are shown in parentheses. Variants with no odds ratio were observed only in high-risk families. All family members were tested for all variants unless no DNA was available. Individuals with no available DNA are indicated.

To determine the potential significance of variants that we identified, we evaluated the segregation pattern of these variants in the relevant pedigrees. We identified potentially detrimental sequence variants in 10 of the 11 pedigrees from which individuals were selected for DNA capture and sequencing. Several of the pedigrees segregated more than one variant, indicating the complexity of the underlying genetics in high-risk ASD pedigrees. Moreover, many of these pedigrees also have CNVs that were identified in our previous work [27]. Adding to the genetic complexity, many of these CNVs also segregate to affected individuals. Five families that demonstrate these complex inheritance patterns are shown here (Figures 2, 3, 4, 5 and 6). Five additional pedigrees with multiple variants are shown in Additional file 7: Figure S6, Additional file 8: Figure S7, Additional file 9: Figure S8, Additional file 10: Figure S9, Additional file 3: Figure S2.

Pedigree 1 (Figure 2) shows a two-generation family co-segregating a missense variant in *RAB11FIP5* (Table 4). This variant is present in the mother and segregates to all three male affected children in the family, and not to the unaffected female child. *RAB11FIP5* has previously been implicated as an ASD risk gene based on its disruption by a translocation observed in a 10 year-old male child with a diagnosis of pervasive developmental disorder not otherwise specified (PDD-NOS) [43]. The variant detected in pedigree 1 results in a P652L substitution. Proline is conserved at this residue in all of the mammalian *RAB11FIP5* genes sequenced to date, suggesting that it is important for protein function. A second individual, with a P652H variant, was detected in our case/control study (Table 3) using our custom microarray. Neither the P652L substitution nor the P652H substitution was observed in the ESP6500, 1,000 genomes project or dbSNP137 databases (Additional file 1: Table S5). Each of these variants was confirmed by Sanger sequencing (See Additional file 5: Figure S4, Additional file 6: Figure S5 for chromatograms). An additional affected individual of non-European descent, and thus not included in the case/control study, also carried the P652H variant (data not shown). The presence of the P652H variant in an additional individual with autism and not in any controls further supports the likelihood of variants in *RAB11FIP5* contributing to autism risk.

Pedigree 2 (Figure 3) is a two-generation family with six affected individuals from two fathers. In this pedigree, five of the six affected individuals inherit a variant resulting in an I26T substitution in *C14orf2*. Two additional sequence variants, one each in the *PDK4* and *SDR39U1* genes, segregate to three and two affected individuals respectively. In addition, a CNV gain (OR = 3.37) described previously [27] is present in one affected individual. The *C14orf2* and *PDK4* variants were maternally inherited, while the *C7orf10* and the CNV were either of paternal origin or occurred as *de novo* variants. Of the variants detected in this family, only the *C7orf10* variant was observed in our case/control study. However, this variant had an odds ratio of 1.62 (95% confidence interval 1.04 to 2.53), suggesting the possibility for a role in autism predisposition in the general population.

Pedigree 3 (Figure 4) also is a two-generation family, with five male children affected with autism. In this pedigree, four of the five affected individuals exhibit maternal inheritance of an F154L variant in the *KLHL6* gene. This A/G nucleotide variant also is found at the first nucleotide of an exon and thus also may affect splicing of the KLHL6 primary transcript. In addition to this variant, three of the five offspring have a paternally inherited D303H missense variant in the *SPATA5L1* gene while two of five also have a maternally inherited P238L change in the *ITPK1* gene. One affected child does not inherit any of these variants. Of interest, none of the variants observed in this small family were observed in any cases or controls in our population study, demonstrating that they are not common autism predisposition loci.

Pedigree 4 (Figure 5) is a six generation family with an ancestor common to all seven male children that are affected with autism. These children all are in the fifth or sixth generations of the pedigree. Linkage analysis was performed previously on this family using Affymetrix 10 K SNP genotype data [29,30], and three regions of significant linkage were identified. These include 3q13.2-q13.31, 3q26.31-q27.3, and 20q11.21-q13.12. These three regions also were identified by haplotype sharing in this study (Figure 5, see Additional file 2: Figure S1C for chromosome 20 haplotype sharing). Four of the seven affected individuals in this family share a P49L variant that is the result of an A/G transition in the *DEFB124* gene on chromosome 20q11.21, consistent with the haplotype sharing that we observed (Additional file 2: Figure S1c) and with the published linkage result. This variant was not observed in cases or controls in our population study. One affected individual in this pedigree does not share the *DEFB124* variant, but instead has a chromosome 3q gain CNV, inherited from his father, that had an odds ratio of 3.74 in our previous study [27]. The elevated odds ratio suggests that this CNV is an autism risk locus.

Two additional affected individuals in pedigree 4 do not carry any variant that we detected in our families. However, as indicated in Figure 5, each of these two individuals is descended from a marry-in spouse with a strong family history of autism, suggesting the possibility of additional undetected variants.

Finally, one affected individual who carries the *DEFB124* variant carries variants in the *HEPACAM2* gene (odds ratio 1.83 in our population study, Table 3), the *AP1G2* gene (odds ratio 1.67, Table 3), the *PYGO1* gene and the *RELN* gene. Neither the *RELN* variant nor the *PYGO1* variant was observed in the case/control study (Table 4). Interestingly, a recent unpublished clinical case identified a heterozygous deletion involving 15 exons of the *RELN* gene in a patient diagnosed with autism and behavior/conduct disorder (Rena Vanzo, personal communication). Homozygous or compound heterozygous mutations in *RELN* are associated with lissencephaly [44,45], but this *RELN* deletion is the first description of an individual with a developmental phenotype that may be due to haploinsufficiency at this locus.

Pedigree 5 (Figure 6) is a four-generation family with nine individuals affected with autism (seven male, two female). Two variants are of particular interest in this family. The first is a CNV including the 5′-flanking region of the *NRXN1* gene. This CNV is inherited from a father who marries into the family in the second generation. This CNV segregates to three of the four descendants of this individual who are diagnosed with autism. An overlapping *NRXN1* CNV was shown in our previous work to have an odds ratio of 14.96 [27], consistent with other publications suggesting a role for *NRXN1* variants in autism, as well as other neurological disorders [46-48]. However, that CNV was shown to extend into the coding region of *NRXN1*, while TaqMan CNV analysis demonstrates that the CNV in pedigree 5 did not (data not shown). Thus, the significance of the *NRXN1* CNV observed in this family is uncertain.

A second variant identified in this family, found on a haplotype shared by all five affected individuals in two branches of the family (Additional file 2: Figure S1c), is a C/T transition in the *AKAP9* gene that results in an R3233C missense substitution. Note that none of the individuals in these two branches of the family carry the *NRXN1* CNV. The *AKAP9* variant was observed in 4/1541 cases and 4/5785 controls in our population study (odds ratio of 3.76, 95% confidence interval 0.94 to 15.03) (Table 3). A second missense variant in the *AKAP9* gene was observed in a single affected individual in a nuclear family (pedigree 6, Additional file 7: Figure S6). This second *AKAP9* variant was not observed in the case/control study (Table 4). The AKAP family of proteins has been suggested to connect different biological pathways that are involved in nervous system development [49].

Pedigree 5 also segregates other variants that are inherited by multiple children affected with autism. One branch of the pedigree segregates a G/C transversion in the *CLMN* gene that results in a P158A missense substitution. This variant yielded an odds ratio of 1.67 (95% confidence interval 0.73 to 3.84) in our case/control study, suggesting that it is an ASD risk allele. A variant in the *ABP1* gene, also the result of a G/C transversion and resulting in an R345P missense substitution, was observed in two affected individuals in a single branch of the family. This variant was maternally inherited and not seen elsewhere in the pedigree. However, this variant was observed in 1/1,541 cases and 0/5,785 controls in our population study (Table 3) and was not observed in the ESP6500, 1,000 Genomes, or dbSNP137 databases (Additional file 1: Table S5), indicating that it may be a very rare ASD risk variant. Finally, a G/T transversion in the *ALX1* gene that results in an R64L missense substitution was paternally inherited by a single individual. This variant also was seen in pedigree 7 (Additional file 8: Figure S7) and was observed multiple times in our population study (27/1,541 cases and 58/5,785 controls) yielding an odds ratio of 1.75 (95% confidence interval 1.11 to 2.77) (Table 3). Expression of this gene also may be increased by a downstream balanced translocation in a family with mental retardation, language delay and microcephaly that segregate with the translocation [50].

Pedigrees 8 to 10 are shown in Additional file 9: Figure S8, Additional file 10: Figure S9, Additional file 3: Figure S2. One of these pedigrees, pedigree 10, carried two haplotypes (chromosomes 2 and 14) segregating to all six affected individuals (Additional file 2: Figure S1a-b). Sequencing of the genes encompassed by these regions did not identify potential causal variants. This could be due to poor sequence coverage of some portions of the genes. However, sequencing of affected individuals in these families did result in the identification of variants that could be autism risk alleles. One of these variants, a G/A transition that result in a Q22X change in the *MOK* gene observed in a single affected individual and inherited from her father, is interesting, as it was observed in our population study and yielded an odds ratio of 3.76 (95% confidence interval 0.53 to 26.67) (Table 3). Other variants in pedigrees 8 to 10 (Additional file 9: Figure S8, Additional file 10: Figure S9, Additional file 3: Figure S2), including some only seen in Utah families and others seen in both families and in our population study also were identified. These variants are included in Table 3 and Table 4.

#### Functional analysis of RAB11FIP5

To uncover the functional consequences of the Rab11FIP5-P652L variant we evaluated binding of Rab11FIP5 to Rab11. Rab11 is a small monomeric GTPase that mediates Rab11FIP5 recruitment to endocytic membranes and is required for Rab11FIP5 function [41]. As shown in Additional file 11: Figure S10A, the P652L substitution did not affect Rab11FIP5 binding to Rab11, nor did it affect its specificity toward the Rab11 GTPase. It was previously shown that Rab11FIP5 forms homodimers and that its ability to dimerize is also required for Rab11FIP5 cellular functions [41]. Thus, we tested the effect of P652L substitution on Rab11FIP5 ability to dimerize. As shown in Additional file 11: Figure S10B, the Rab11FIP5-P652L mutant was still able to form dimers. Consistent with *in vitro* binding data, FIP5-GFP-P652L endocytic localization in HeLa cells was also not affected (Additional file 11: Figure S10).

It is now well established that Rab11FIP5 functions by regulating endocytic recycling [51]. To that end, we next tested whether Rab11FIP5-P652L has an effect on recycling of transferrin receptors in HeLa cells and found that the P652L substitution did not alter recycling (Additional file 11: Figure S10H). Thus, so far we were unable to find any functional consequences of *Rab11FIP5*-P652L substitution, suggesting that core Rab11FIP5 properties are not affected. Further studies will be needed to uncover the kinetic or binding defects of Rab11FIP5 variants, especially within the context of neuronal cells.

## Discussion

Numerous studies have implicated genetics in the etiology of ASDs, and recent results have implicated hundreds of genes and chromosomal regions as candidate ASD predisposition loci. These include chromosomal rearrangements as well as sequence variants. None of these candidates accounts for more than a small portion of ASD cases, and it is clear, based on the apparent complexity of autism genetics, that many genes are still undiscovered. In multiplex ASD pedigrees, it has been suggested that inherited genetic variants are more likely to be responsible, while in simplex families *de novo* variants may be more important [8,16].

We used a discovery/validation strategy based on identifying inherited genetic variants in two- to nine-generation ASD families, followed by a case/control analysis of those variants in DNA samples from unrelated children with autism and children with normal development, to identify putative familial ASD predisposition genes. Using haplotype analysis we identified shared genomic segments within the families, and used DNA sequencing and CNV analysis to identify potential causal mutations on those haplotypes. We also followed this with a large case/control study to determine if any of the variants we identified might play a role in the general population of individuals with ASD.

We showed previously that identification of CNVs in a family-based discovery cohort could identify copy number variants relevant to the general ASD population [27]. In this paper, we applied the same strategy to the family-based identification of DNA sequence variants, and also follow up on our CNV analysis by providing information regarding segregation of some of these CNVs in our high-risk ASD families.

We identified 39 SNPs that are likely to affect protein function that have segregation patterns and ASD case allele frequencies suggestive of a role in ASD predisposition. Thirty-one of these variants result in non-conservative amino acid substitutions, five are predicted to affect splicing (three of these are predicted to affect both splicing and protein coding), and three introduce premature termination codons. Two variants were identified in the *AKAP9* gene and the *JMJD7* gene (or the *JMJD7-PLA2G4B* fusion gene), and two different variants were identified that affect the same amino acid residue in the *RAB11FIP5* gene; so collectively, these SNPs identify 36 potential ASD risk genes.

With the exception of two-generation families, and consistent with our haplotype sharing results, no sequence variants or CNVs implicated as ASD predisposition loci segregate to all affected individuals in a pedigree. This is consistent with previous genetic studies, which to date have been unable to demonstrate segregation of a single ASD risk locus in an extended family (for example see [52]). In our pedigree 5 (Figure 6), two independent risk variants, a single nucleotide variant in *AKAP9* and a deletion CNV in or near *NRXN1*, segregate to different branches of the family. Other risk variants also are found in individuals with ASD in this family, including two sequence variants with odds ratios greater than 1.5 in our population study. These results suggest that even in extended families that might be predicted to be segregating a single risk allele with reduced penetrance, multiple risk alleles in different ASD predisposition loci may be necessary. The results further suggest that use of specific inheritance models when evaluating autism genetics in large families should be approached with caution. Further, given our sequencing results and CNVs, the data suggest that a two-pronged approach, involving both DNA sequencing and microarray-based CNV analysis, may be necessary for a complete genetic diagnosis of children with ASDs.

Eleven of the putative autism risk variants that we identified in our high-risk families are further supported by data from our case/control study. Three of these variants each were seen in a single ASD case (out of 1,541 total cases) and in none of 5,785 controls. Familial variants that we detected in eight additional genes are more common in ASD cases than in controls, and each has an odds ratio greater than 1.5. Although these variants are rare (all have frequencies of < 0.01 in our case/control study), their identification in affected individuals in our ASD families and their increased prevalence in unrelated affected individuals support their role as potential ASD risk loci.

Several intriguing observations resulted from an extensive literature review of the functions and mechanistic actions of each of these 36 genes and their encoded proteins. A number of the genes have been previously linked to autism or other neurological disorders or have known neurological functions (Table 5) (11 out of 36 genes, or 31%). The functions of several other genes belong to pathways often cited as having relevance to autism. These include genes encoding proteins with immunological functions (inflammatory response), and genes encoding proteins important for energy metabolism and mitochondrial function. These groups account for 19 of the 36 genes on the list (53%). Other genes have as yet unexplored functions, can only be linked to functions based on sequence similarity, or have scattered roles in many other cellular or organismal processes, such as cell cycle control, angiogenesis, protein degradation, or metalloproteinase activity.

**Table 5:** Biological functions/pathways of genes with variants found in children with ASDs

**Function**	**Gene names**	**References**
Previously associated with autism	*TRPM1*, *RAB11FIP5*, *AKAP9*, *SCN3A*	[27,43,49,53]
Previously associated with neurological disorder (other than autism)	*RELN* (autosomal recessive lissencephaly), *ALX1* (facial clefting, micropthalmia), *CCDC85C* (seizures), *EPB41L1* (intellectual disability)	[44,45,50,54-56]
Neural function	*ITPK1*, *CLMN*, *PRTG*	[57-59]
Mitochondrial function	*PLA2G4B*, *c7orf10*, *PDK4*, *C14orf2*	[60-63]
Inflammatory response/Immune function	*DEFB124*, *BPI*, *RNF31*, *IRF9*	[64-67]

The incidence/prevalence of autism has been reported to be as high as 1 in 38 children [68], while the estimated prevalence in the US is reported to be approximately 1 in 88 [69]. Multiple factors, including genetic, epigenetic and environmental, are thought to be necessary for autism development. As with previous work involving familial autism, we found that no single gene variant described here can account for every case of autism in our pedigrees. This may reasonably be expected of a multigenic disorder with high prevalence in the population. It also is consistent with the high likelihood that autism represents a number of different conditions, at least at the genetic level, all under the same phenotypic umbrella called autism. Several variants that we identified, however, represent excellent candidates for significant risk factors for autism based on their known functions and the currently understood molecular pathways involved in autism. We discuss the most compelling cases below.

### RAB11FIP5

RAB11FIP5 is a member of a family of scaffolding proteins for the RAS GTPase, Rab11. Specifically, RAB11FIP5 has been characterized as a key player in apical endosome recycling, plasma membrane recycling and transcytosis [70,71]. We identified a P652L variant in three affected siblings in a family of six members, in which the mother is an unaffected P652L carrier. An additional variant resulting in a P652H substitution also was detected in 1/1,541 Caucasian ASD cases and 0/5,785 Caucasian children with normal development (Table 3). These variants modify a conserved proline within the C-terminus of RAB11FIP5.

Heterozygous disruption of *RAB11FIP5* was observed previously in a ten year old boy with a balanced translocation (46, XY, t(2;9)(p13;p24)) that disrupts only the *RAB11FIP5* gene [43]. This individual has a clinical diagnosis of PDD-NOS, an autism spectrum disorder. This translocation led the authors to suggest that haploinsufficiency of RAB11FIP5 contributes to the subject’s ASD. RAB11FIP5 works closely in conjunction with RAB11, and its presence has been detected in both presynaptic and post-synaptic densities where Rab11 plays a key role in determining synaptic strength in long-term depression [72], regulates norepinephrine transporter trafficking [73], carries out synaptic glutamate receptor recycling [74], and regulates dendritic branching in response to BDNF [75,76]. All of these functions have been suggested to be significant contributors to the etiology of ASDs [77,78] and further support the role of mutations in *RAB11FIP5* as ASD risk alleles.

### AKAP9

AKAP9 is a member of a family of over 50 proteins that serve as scaffolding partners for PKA, its effectors, and phosphorylation targets. *AKAP9*, also known as Yotiao, is chiefly expressed in the heart and brain, where the encoded protein serves as a scaffold for PKA, protein phosphatase I, NMDA receptors, the heart potassium channel subunit KCNQ1, IP3R1, and specific isoforms of adenylyl cyclase [79-83]. The subcellular localization and assembly of these multimeric protein scaffolds, mediated by AKAPs, are thought to be essential for function, since disruption of the interaction between the AKAP and its effectors leads to a loss of activity. In the case of KCNQ1, loss of interaction between AKAP9 and KCNQ1 leads to a potentially fatal heart condition, long-QT syndrome, which also arises in cases with loss of function mutations in *KCNQ1* itself [84].

We identified two variants in the *AKAP9* gene. These variants result in R3233C and R3832C substitutions in the encoded protein. These two variants were coincident with autism and were found in two unrelated extended ASD pedigrees (Figure 6, Additional file 7: Figure S6). The R3233C variant was additionally found in our case/control study. A recent meta-study of the genes identified from the five major autism GWAS studies and autism candidate genes arising from alternative methodologies, such as large scale CNV studies, placed AKAPS as a central, integral gene family linking many of the pathways identified by bioinformatics [49]. Given its role in localizing PKA, adenylyl cyclase isoforms and NMDAR in the postsynaptic scaffold, AKAP9 represents a protein that, like its better-characterized counterpart AKAP5, could function in synaptic transmission and plasticity, glutamatergic receptor function regulation and recycling, and dendritic spine morphology [85].

It is notable that two of the genes (*MOK*, *TRPM1*) containing potential ASD risk alleles were partially or completely encompassed by risk CNVs observed in our previous study [27]. This suggests that the same genes may be affected by different genetic mechanisms with the same or similar phenotypic result. The CNVs containing these genes were both copy number losses. The *MOK* sequence variant described here was a nonsense change, while the *TRPM1* variant was a missense change. These results are consistent with the *MOK* and *TRPM1* effects being due to haploinsufficiency at these two loci.

Our data demonstrate the complex nature of autism genetics. Although the heritability for autism is quite high, our data show that numerous genetic variants may confer risk to ASD even in a single family. This finding is consistent with the results of a whole genome sequencing study that used both a recessive model and model independent analyses to identify several potential ASD risk variants in an ASD family with two affected individuals [86]. Consistent with the large number of potential ASD risk genes identified to date, none of the genes identified in this single multiplex ASD family overlapped with the genes identified in our study. Our study adds to this complexity by identifying sequence variants in regions of haplotype sharing in 30 high-risk ASD families of two to six generations. Our data further demonstrate that in very large multi-generation families, the likelihood of additional risk variants entering the family from individuals who marry into the pedigree is high. These results are consistent with the prevalence of autism in the population and the high heritability for autism seen in numerous studies. Future studies will be necessary to characterize the biological roles that the variants we identified play in the complex set of disorders that comprise ASD.

## Conclusions

Our study is the first to use an empirical approach to identify shared genomic segments, followed by sequence variant detection, to identify potential ASD risk variants in a large set of autism families. We identified 39 DNA sequence variants in 36 genes that may represent ASD risk genes. Eleven of these variants may also be risk variants in unrelated ASD cases, as they were shown to have odds ratios greater than 1.5 in a large ASD case/control population. Twenty-eight of these variants were observed only in high-risk ASD families in our study. These very rare sequence changes also may identify additional ASD risk genes. Segregation patterns demonstrate that in multi-generation ASD families, multiple genetic variants, including CNV and SNP risk variants, may contribute to ASD risk.

## Abbreviations

ASD: Autism spectrum disorder; CNV: Copy number variant; SNP: Single nucleotide polymorphism; NCBI: National Center for Biotechnology Information; UPDB: Utah Population Database; ADI-R: Autism Diagnostic Interview-Revised; ADOS: Autism Diagnostic Observation Schedule; CHOP: The Children’s Hospital of Philadelphia; PCA: Principal component analysis; MAF: Minor allele frequency; HWE: Hardy-Weinberg equilibrium; GST: Glutathione S-transferase; SDS: Sodium dodecyl sulfate; PDD-NOS: Pervasive developmental disorder not otherwise specified.

## Competing interests

GBC and CGL are paid employees of Golden Helix Inc., which derives commercial revenue from the SNP & Variation Suite software used for data analysis for this publication. NM, CHH and MFL have stock options and patent applications with Lineagen, Inc. MFL also is an unpaid scientific advisor for Lineagen, Inc. CHH is an employee of, and KH is a former employee of, Lineagen, Inc. The remaining authors declare no competing interests. Financial support from Lineagen, Inc. does not alter our adherence to all the *Molecular Autism* policies on sharing data and materials.

## Authors’ contributions

NM designed experiments, performed DNA sequencing and molecular analysis, analyzed data and contributed to writing of the manuscript. CHH designed experiments, oversaw data analysis, and wrote much of the manuscript. LB performed molecular confirmation assays on high-risk family sequence variants and designed and performed melting curve assays for SNP confirmation. JS and BO performed sequence analysis for identifying sequence variants in our high-risk autism families. TL performed haplotype sharing analysis on our high-risk ASD families. TV handled all aspects of autism family DNA sample QC and tracking. DH contributed to experimental design. JTG supervised all data release and performed QC on all data generated at CHOP. RP supervised sequencing work at CHOP and oversaw data quality. CK and KT generated and helped analyze most of the array-based SNP data generated at CHOP. FW oversaw all aspects of DNA sample handling of CHOP samples. FGO performed Sanger sequencing and analysis to confirm variants in CHOP samples. KH evaluated the biological roles of all genes with variants and contributed to writing of the manuscript. GBC performed data QC and analysis, and contributed to writing of the manuscript. DL and RP performed and evaluated *RAB11FIP5* functional assays and wrote portions of the manuscript. CGL, HH and MFL, oversaw lab and analytical operations, aided significantly in experimental design and setup, contributed to the writing of the manuscript and approved the final version of the manuscript. All authors read and approved the final manuscript.

## Supplementary Material

Additional file 1: Table S1 Pedigree structure. **Table S2.** Literature ASD risk genes sequenced. **Table S3.** Primers for variant amplification and sequencing. **Table S4** List of all confirmed variants. **Table S5.** Analysis of potential risk variants. Click here for file

Additional file 2: Figure S1 Haplotype sharing in high-risk autism pedigrees. The figures show a graphic representation of haplotype sharing among affected individuals in a pedigree, created using the HapShare program. The X-axis represents chromosomal coordinates for the designated chromosomes. The Y-axis represents various combinations of haplotype sharing among affected individuals in the pedigree, listed arbitrarily by iteration number. The lowest value on the Y-axis represent sharing among all N affected individuals in the pedigree, and where all N individuals share, there is only one possible combination. With lower degrees of sharing there are more possibilities. For example, in pedigree 10 with six affected individuals, there is only one possible way for all six to share the same haplotype. Where only five of six share the haplotype, there are six different ways to get this result, with each of the six affected individuals being excluded from sharing in each of the six iterations shown. Red indicates sharing among N out of N affected individuals in the pedigree, with other colors representing lower degrees of sharing. Panel a) two regions of chromosome 2 shared by all six affected individuals in pedigree 10; panel b) sharing among all six affected individuals in pedigree 10 of a chromosome 14 region; panel c) sharing among five of eight affected individuals on chromosome 7 in pedigree 5 and sharing among four of seven affected individuals on chromosome 20 in pedigree 4. The variants found on these haplotypes are indicated by the gene names in the figure. Note that the chromosome 7 region identified in pedigree 5 as being shared among eight affected individuals was later shown not to be shared by an additional affected family member, resulting in a final count of sharing among five of nine affected individuals. Click here for file

Additional file 3: Figure S2 Segregation of sequence variants in *SCN3A* and *OIP5* and CNVs involving *LINGO2* in pedigree 10. Pedigree 10 has 6 affected male siblings. The female sibling in the lowest generation has trisomy 21 and includes some features of autism. The *LINGO2* loss CNV was shown to have an odds ratio of 3.74 in our case/control study, while the *LINGO2* gain CNV did not have a clinically relevant odds ratio in the broad ASD population. The *SCN3A* sequence variant was not observed in our case/control study while the *OIP5* variant yielded an odds ratio of 2.25. Pedigree symbols are described in the legend for Figure 2. Sequence variants identified in the family are shown in the black boxes. All family members with DNA available were tested for all variants. Click here for file

Additional file 4: Figure S3 SNP genotype clusters. Genotype clusters for all SNPs observed in the case/control study (Table 3) are shown. Click here for file

Additional file 5: Figure S4 Sanger sequence confirmation of variants in the *RAB11FIP5, AUP1, SCN3A, ATP11B, KLHL6, C7orf10, AKAP9, HEPACAM2, PDK4, RELN, ABP1, ALX1, AP1G2, DCAF11, RNF31, IRF9, SDR39U1* and *PRKD1* genes. Heterozygous positions are indicated by the blue line in the center of each panel. Click here for file

Additional file 6: Figure S5 Sanger sequence confirmation of variants in the *SEC23A, ITPK1, CLMN, CCDC85C, MOK, C14orf2, TRPM1, FMN1, PGBD4, OIP5, JMJD7, JMJD7-PLA2G4B, CASC4, SPATA5L1, PYGO1, PRTG, NUDT7, DEFB124* and *EPB41L1* genes. Heterozygous positions are indicated by the blue line in the center of each panel. Click here for file

Additional file 7: Figure S6 Segregation of a second AKAP9 variant in a small pedigree. Pedigree 6 has a single affected child. Pedigree symbols are described in the legend for Figure 2. A link between this pedigree and other high-risk autism pedigrees is indicated by blue boxes. Sequence variants identified in the family are shown in the black boxes. Odds ratios for the variants observed in the case/control study are shown in parentheses. Variants with no odds ratio were observed only in high-risk families. All family members were tested for all variants unless no DNA was available. Individuals with no available DNA are indicated. Click here for file

Additional file 8: Figure S7 Segregation of an *ALX1* variant in a small two-generation pedigree. Pedigree 6 has two siblings affected with autism. A single *ALX1* variant is shared by both siblings. A link between this pedigree and another high-risk autism pedigree is indicated by the blue box. Pedigree symbols are described in the legend for Figure 2. Sequence variants identified in the family are shown in the black boxes. Odds ratios for the variants observed in the case/control study are shown in parentheses. Variants with no odds ratio were observed only in high-risk families. All family members were tested for all variants. Click here for file

Additional file 9: Figure S8 Multigeneration pedigree with multiple sequence variants and overlapping loss and gain copy number variants. Pedigree 8 has 5 affected male children. Potential causal variants in this family do not segregate to more than one affected individual. CNVs identified in 4 individuals [27] are shown in red boxes. Pedigree symbols are described in the legend for Figure 2. Sequence variants identified in the family are shown in the black boxes. Odds ratios for the variants observed in the case/control study are shown in parentheses. Variants with no odds ratio were observed only in high-risk families. All family members were tested for all variants unless no DNA was available. Individuals with no available DNA are indicated. Click here for file

Additional file 10: Figure S9 Segregation of two sequence variants in a two-generation pedigree. Pedigree 9 has three affected female siblings. Pedigree symbols are described in the legend for Figure 2. Sequence variants identified in the family are shown in the black boxes. All family members were tested for all variants. Click here for file

Additional file 11: Figure S10 Effects of RAB11FIP5 P652L on RAB11 binding. (A) Wild- type of P652L mutant FIP5(490–653) was incubated with either various GST-tagged Rabs or GST-tagged FIPs. Beads were then washed and bound FIP5(490–653) eluted with 1% SDS. Eluates were then analyzed by immunoblotting with anti-Rab11FIP5 antibodies. (B-G) HeLa cells were transduced with either wild-type FIP5-GFP (A and D) or FIP5-GFP-P652L (E and G). Cells were then fixed and stained with anti-transferrin receptor antibodies (C, D, F and G). D and E are merged images, with yellow representing the extent of overlap between Rab11FIP5 and transferrin receptor. (H) HeLa cells expressing either FIP5-GFP or FIP5-GFP-P652L were incubated with 1 μg/ml of transferrin-Alexa488. Cells were then washed and incubated in serum-supplemented media varying amount of time. Cell-associated (not recycled) transferrin-Alexa488 was measured using flow cytometry. Data shown are the means of two independent experiments. Click here for file
