# Supplementary figures and images for: Identification of rare DNA sequence variants in high-risk autism families and their prevalence in a large case/control population
Source: Mol Autism. 2014 Jan 27;5:5. doi: 10.1186/2040-2392-5-5 (PMC4098669; doi:10.1186/2040-2392-5-5)

## Supplementary Figure 3

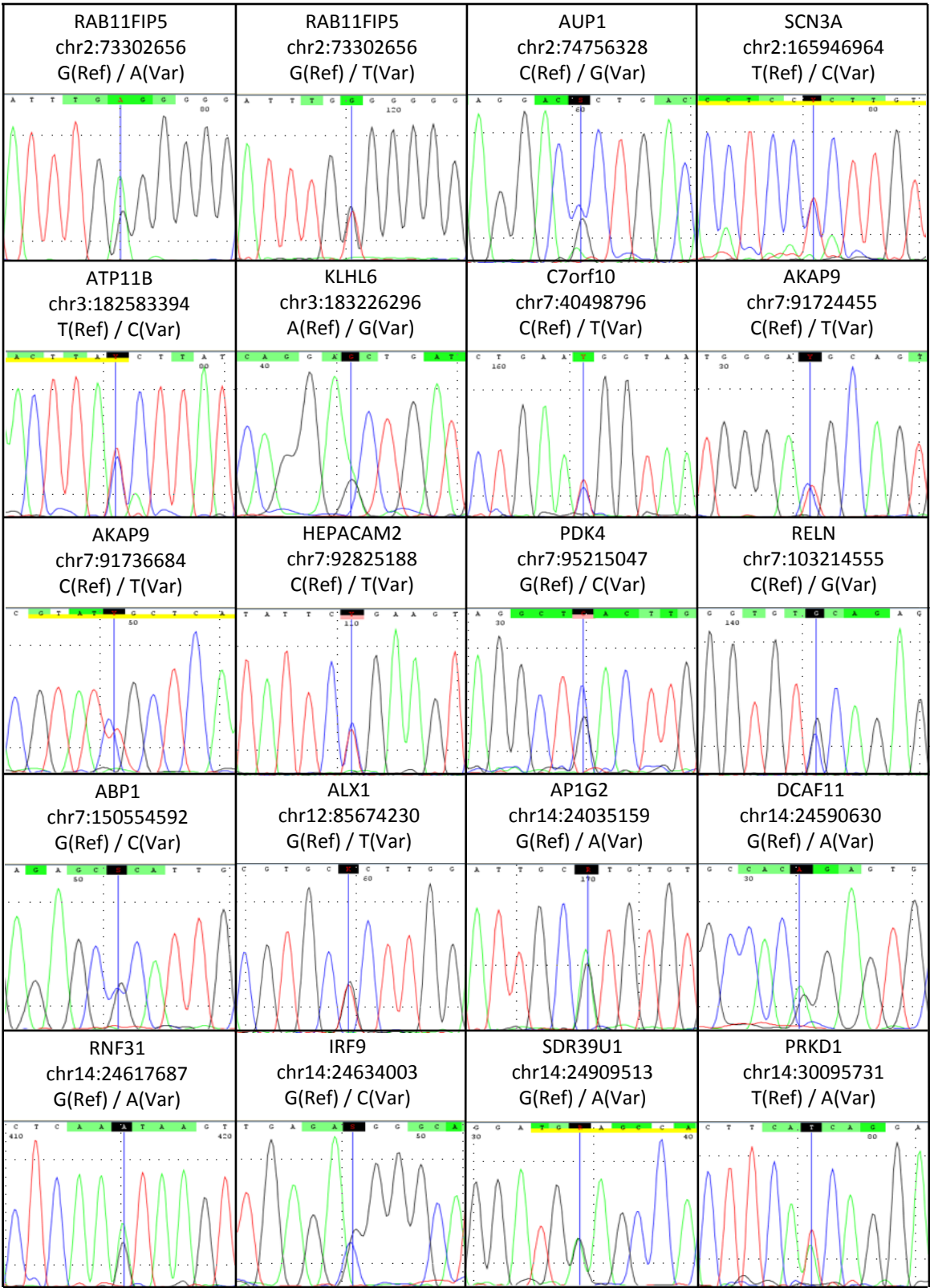

Supplement: Additional file 2: Figure S1 — Haplotype sharing in high-risk autism pedigrees. The figures show a graphic representation of haplotype sharing among affected individuals in a pedigree, created using the HapShare program. The X-axis represents chromosomal coordinates for the designated chromosomes. The Y-axis represents various combinations of haplotype sharing among affected individuals in the pedigree, listed arbitrarily by iteration number. The lowest value on the Y-axis represent sharing among all N affected individuals in the pedigree, and where all N individuals share, there is only one possible combination. With lower degrees of sharing there are more possibilities. For example, in pedigree 10 with six affected individuals, there is only one possible way for all six to share the same haplotype. Where only five of six share the haplotype, there are six different ways to get this result, with each of the six affected individuals being excluded from sharing in each of the six iterations shown. Red indicates sharing among N out of N affected individuals in the pedigree, with other colors representing lower degrees of sharing. Panel a) two regions of chromosome 2 shared by all six affected individuals in pedigree 10; panel b) sharing among all six affected individuals in pedigree 10 of a chromosome 14 region; panel c) sharing among five of eight affected individuals on chromosome 7 in pedigree 5 and sharing among four of seven affected individuals on chromosome 20 in pedigree 4. The variants found on these haplotypes are indicated by the gene names in the figure. Note that the chromosome 7 region identified in pedigree 5 as being shared among eight affected individuals was later shown not to be shared by an additional affected family member, resulting in a final count of sharing among five of nine affected individuals. [file 2040-2392-5-5-S2.pdf]

## Supplementary Figure 4

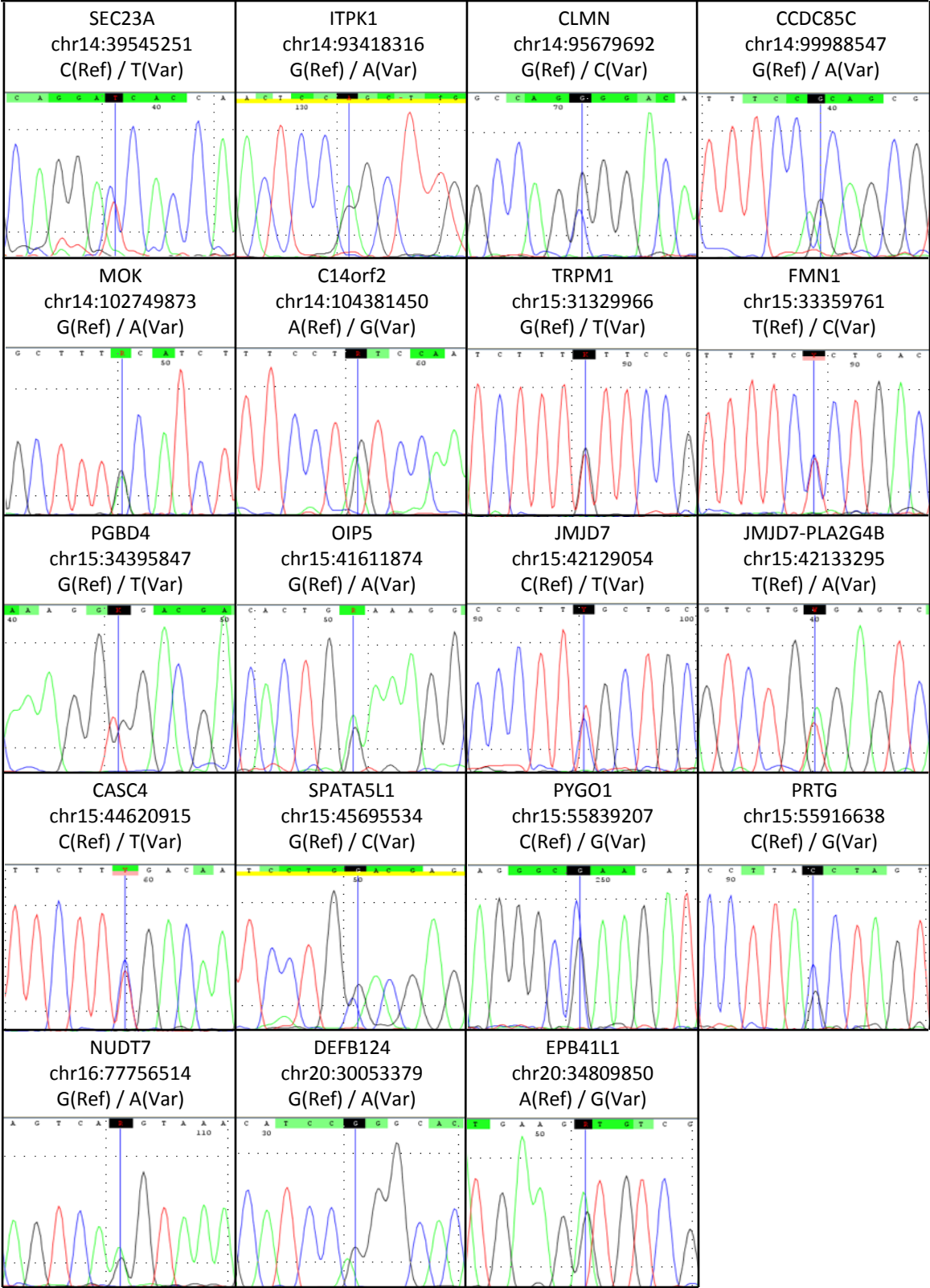

Supplement: Additional file 3: Figure S2 — Segregation of sequence variants in SCN3A and OIP5 and CNVs involving LINGO2 in pedigree 10. Pedigree 10 has 6 affected male siblings. The female sibling in the lowest generation has trisomy 21 and includes some features of autism. The LINGO2 loss CNV was shown to have an odds ratio of 3.74 in our case/control study, while the LINGO2 gain CNV did not have a clinically relevant odds ratio in the broad ASD population. The SCN3A sequence variant was not observed in our case/control study while the OIP5 variant yielded an odds ratio of 2.25. Pedigree symbols are described in the legend for Figure 2. Sequence variants identified in the family are shown in the black boxes. All family members with DNA available were tested for all variants. [file 2040-2392-5-5-S3.pdf]

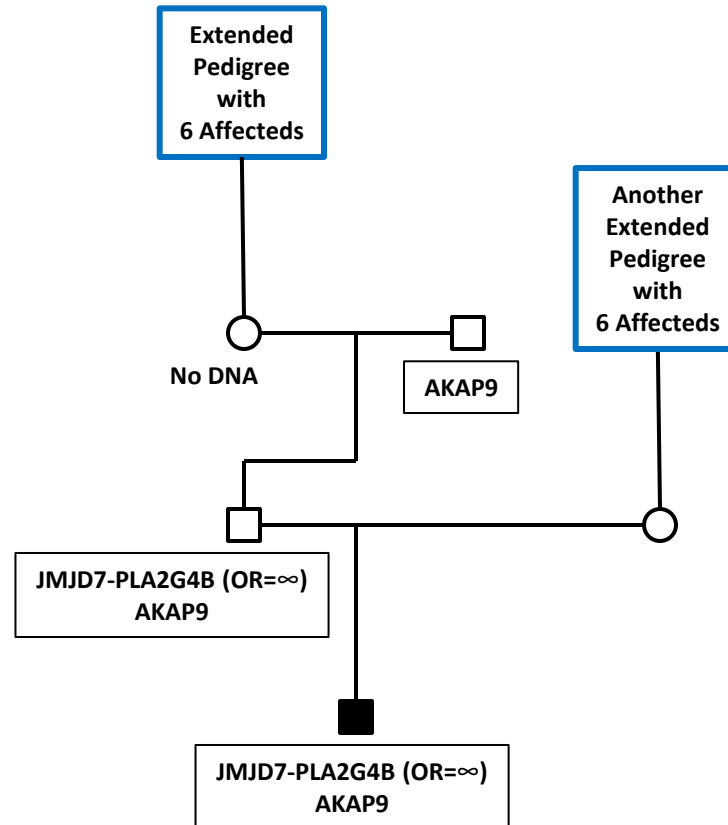

Supplement: Additional file 4: Figure S3 — SNP genotype clusters. Genotype clusters for all SNPs observed in the case/control study (Table 3) are shown. [file 2040-2392-5-5-S4.pdf]

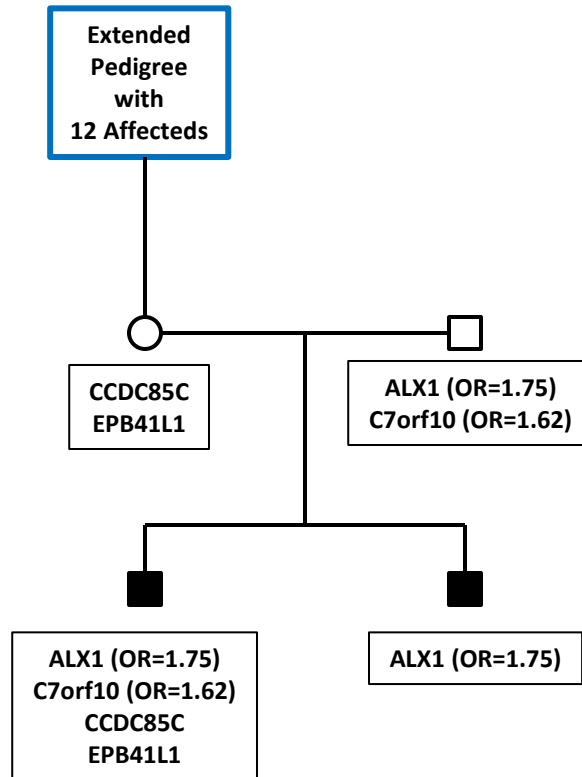

Supplement: Additional file 5: Figure S4 — Sanger sequence confirmation of variants in the RAB11FIP5, AUP1, SCN3A, ATP11B, KLHL6, C7orf10, AKAP9, HEPACAM2, PDK4, RELN, ABP1, ALX1, AP1G2, DCAF11, RNF31, IRF9, SDR39U1 and PRKD1 genes. Heterozygous positions are indicated by the blue line in the center of each panel. [file 2040-2392-5-5-S5.pdf]

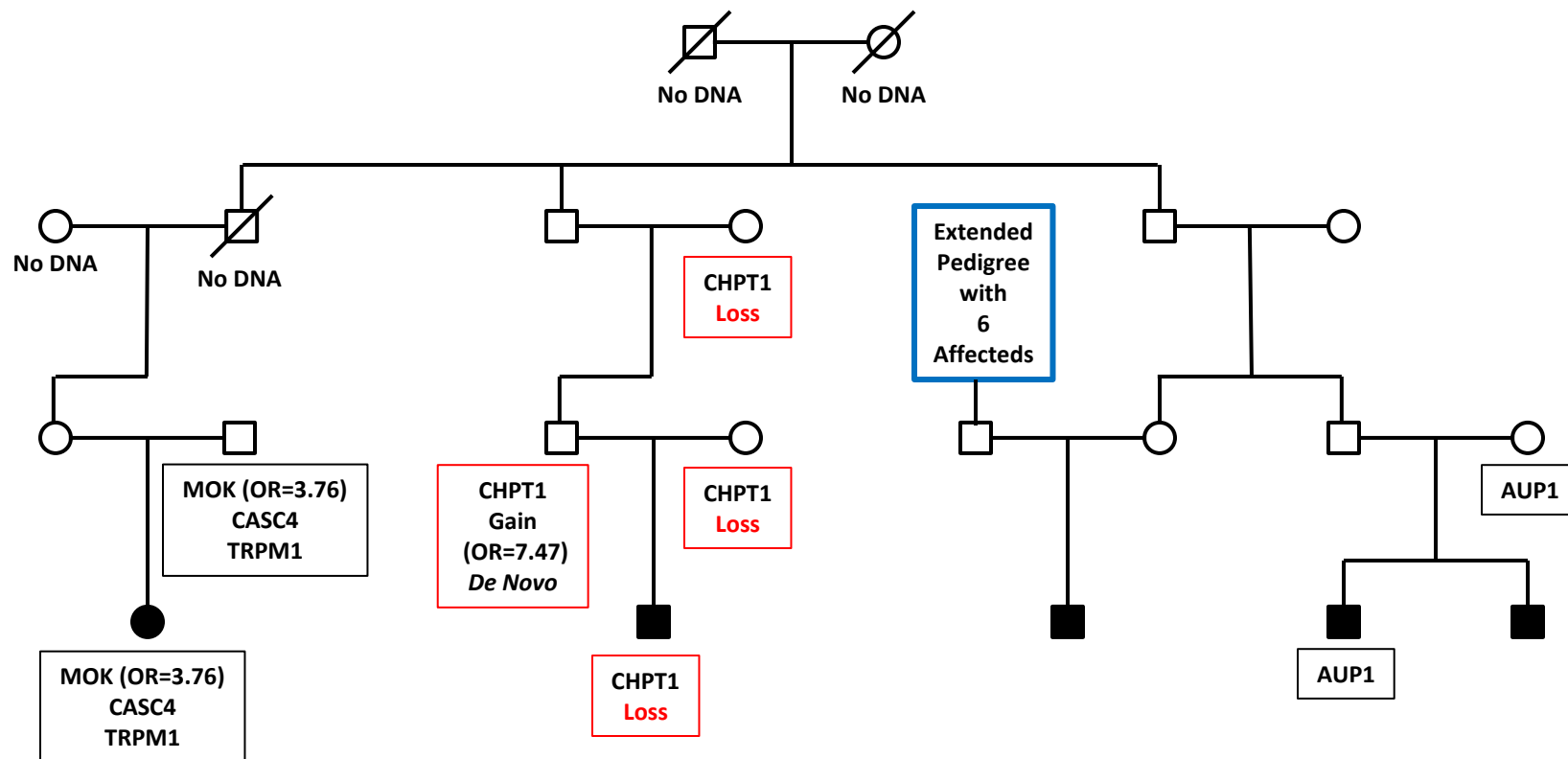

Supplement: Additional file 6: Figure S5 — Sanger sequence confirmation of variants in the SEC23A, ITPK1, CLMN, CCDC85C, MOK, C14orf2, TRPM1, FMN1, PGBD4, OIP5, JMJD7, JMJD7-PLA2G4B, CASC4, SPATA5L1, PYGO1, PRTG, NUDT7, DEFB124 and EPB41L1 genes. Heterozygous positions are indicated by the blue line in the center of each panel. [file 2040-2392-5-5-S6.pdf]

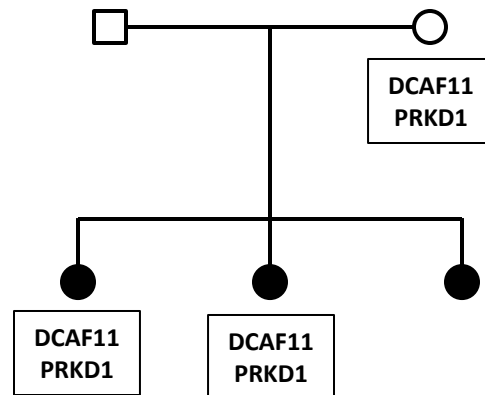

Supplement: Additional file 7: Figure S6 — Segregation of a second AKAP9 variant in a small pedigree. Pedigree 6 has a single affected child. Pedigree symbols are described in the legend for Figure 2. A link between this pedigree and other high-risk autism pedigrees is indicated by blue boxes. Sequence variants identified in the family are shown in the black boxes. Odds ratios for the variants observed in the case/control study are shown in parentheses. Variants with no odds ratio were observed only in high-risk families. All family members were tested for all variants unless no DNA was available. Individuals with no available DNA are indicated. [file 2040-2392-5-5-S7.pdf]

A

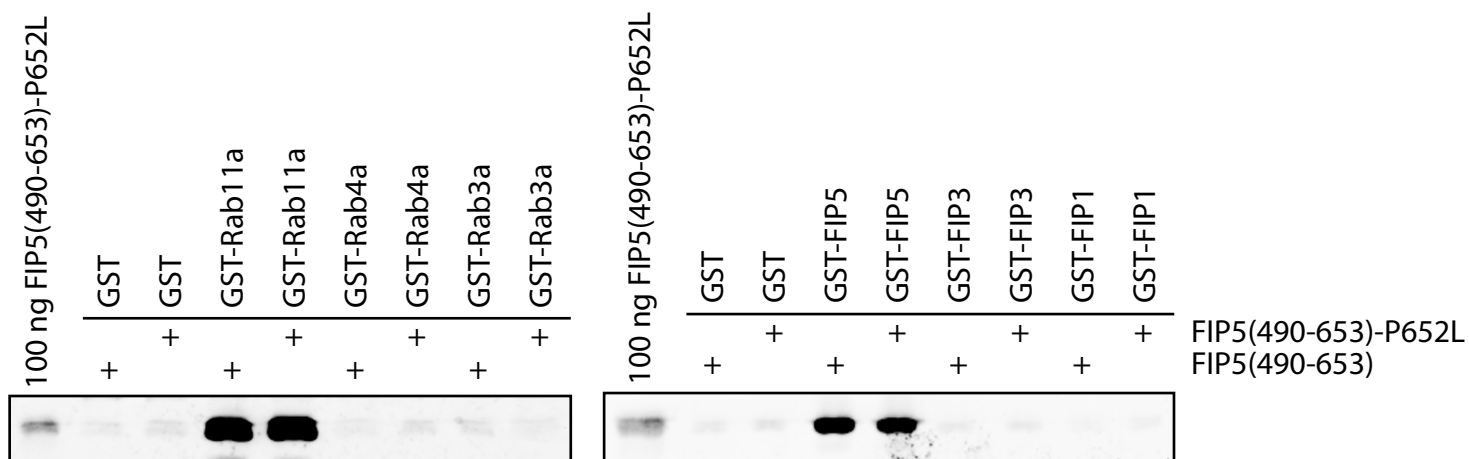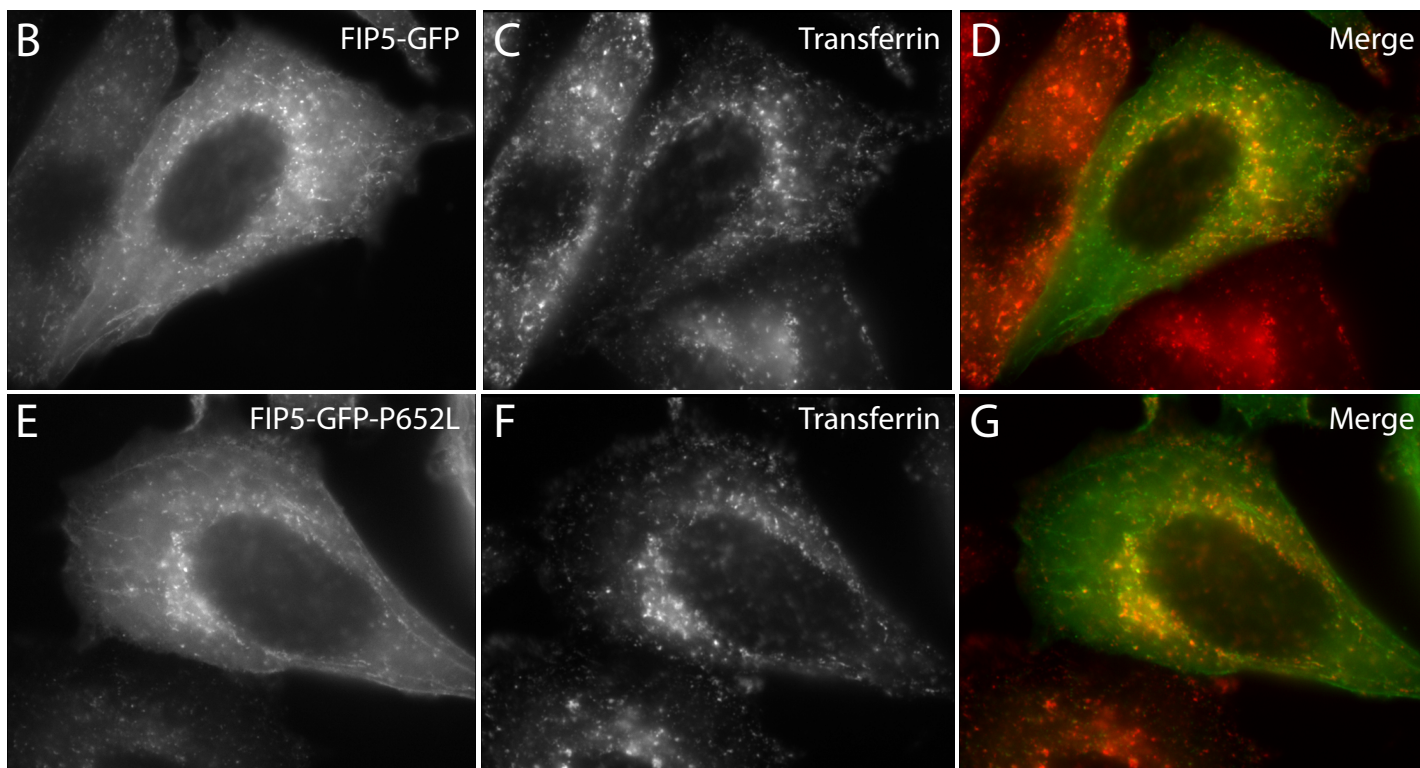

H

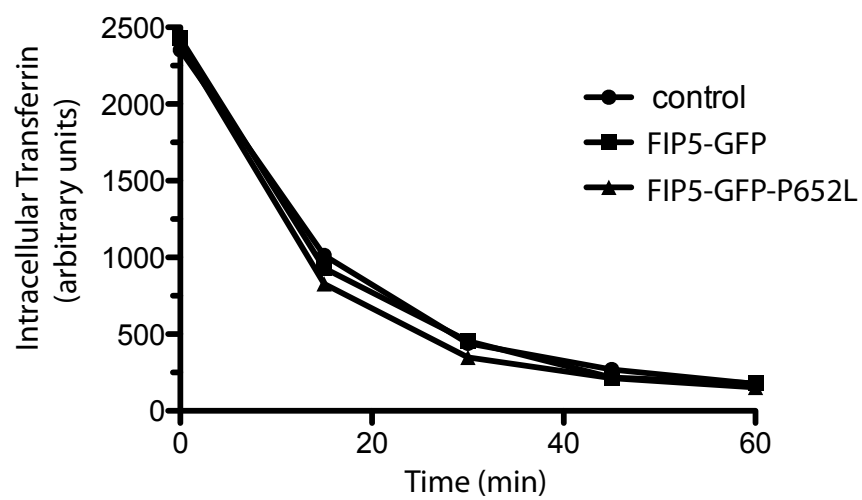

Supplement: Additional file 8: Figure S7 — Segregation of an ALX1 variant in a small two-generation pedigree. Pedigree 6 has two siblings affected with autism. A single ALX1 variant is shared by both siblings. A link between this pedigree and another high-risk autism pedigree is indicated by the blue box. Pedigree symbols are described in the legend for Figure 2. Sequence variants identified in the family are shown in the black boxes. Odds ratios for the variants observed in the case/control study are shown in parentheses. Variants with no odds ratio were observed only in high-risk families. All family members were tested for all variants. [file 2040-2392-5-5-S8.pdf]
